# Strategies for combating plant salinity stress: the potential of plant growth-promoting microorganisms

**DOI:** 10.3389/fpls.2024.1406913

**Published:** 2024-07-15

**Authors:** Biswa R. Acharya, Satwinder Pal Gill, Amita Kaundal, Devinder Sandhu

**Affiliations:** ^1^ US Salinity Laboratory, USDA-ARS, Riverside, CA, United States; ^2^ College of Natural and Agricultural Sciences, University of California Riverside, Riverside, CA, United States; ^3^ Plants, Soils, and Climate, College of Agricultural and Applied Sciences, Utah State University, Logan, UT, United States

**Keywords:** climate change, glycophyte, ion toxicity, osmotic stress, PGPMs, salinity tolerance, salt stress

## Abstract

Global climate change and the decreasing availability of high-quality water lead to an increase in the salinization of agricultural lands. This rising salinity represents a significant abiotic stressor that detrimentally influences plant physiology and gene expression. Consequently, critical processes such as seed germination, growth, development, and yield are adversely affected. Salinity severely impacts crop yields, given that many crop plants are sensitive to salt stress. Plant growth-promoting microorganisms (PGPMs) in the rhizosphere or the rhizoplane of plants are considered the “second genome” of plants as they contribute significantly to improving the plant growth and fitness of plants under normal conditions and when plants are under stress such as salinity. PGPMs are crucial in assisting plants to navigate the harsh conditions imposed by salt stress. By enhancing water and nutrient absorption, which is often hampered by high salinity, these microorganisms significantly improve plant resilience. They bolster the plant’s defenses by increasing the production of osmoprotectants and antioxidants, mitigating salt-induced damage. Furthermore, PGPMs supply growth-promoting hormones like auxins and gibberellins and reduce levels of the stress hormone ethylene, fostering healthier plant growth. Importantly, they activate genes responsible for maintaining ion balance, a vital aspect of plant survival in saline environments. This review underscores the multifaceted roles of PGPMs in supporting plant life under salt stress, highlighting their value for agriculture in salt-affected areas and their potential impact on global food security.

## 1 Introduction

Climate change poses a formidable challenge to global agricultural productivity, with agriculture being particularly vulnerable to shifts in weather patterns and climate conditions. A persistent increase in the average global temperatures has been recorded in recent years, posing significant challenges to agricultural productivity, food security, and environmental sustainability (Siegel, 2021). Climate change not only is limited to increasing average global temperature but also includes erratic rainfall patterns, heat waves, droughts, and flash floods, all of which adversely affect soil and water resources, agricultural workers, and rural communities (https://www.epa.gov/climateimpacts/climate-change-impacts-agriculture-and-food-supply). Regions that rely heavily on agriculture, such as South Asian countries, are particularly impacted by these climate-related challenges (Rhaman et al., 2022). The global population is projected to exceed 10 billion within the next 50 years (Glick, 2014), significantly increasing the demand for food production and placing additional strain on existing agricultural systems (Alexandratos, 2005; Cheeseman et al, 2016). This surge in population presents the dual challenges of boosting agricultural productivity amidst increasingly worsening environmental conditions. Among these challenges, drought and salt stress are the two major abiotic stressors that significantly reduce crop yields and threaten food security and livelihoods (Cheeseman et al, 2016).

Innovative strategies should be developed to address the food security crisis and meet the demand of the projected growing population in the climate change-induced environmental stresses (Wang et al., 2020). The approaches being used currently, including genetically modified organisms, have shown promise in mitigating the impact of drought and salinity stress (Askari and Pepoyan, 2012; Liang, 2016; Raza et al., 2023). However, regulatory constraints and environmental concerns are hurdles to widespread adoption and spread. Other candidate approaches include agronomic management practices (Majeed and Siyyar, 2020) and soil amendments (Bello et al., 2021). Organic amendments like biochar, bio-fertilizer, vermicompost, and vermiwash can improve the salinity tolerance of agricultural plants, leading to increased yields (Hoque et al., 2022). Additionally, seed priming and exogenous application of growth regulators can alleviate salt stress impacts in plants at various stages of development from germination to maturity (Tania et al., 2022). However, these approaches have limited effectiveness under harsh conditions, can be expensive, may vary in efficacy with different crop species, and may have environmental impacts.

Beneficial microorganisms colonize plants’ rhizospheres or inside tissues, promoting growth, improving nutrient uptake, and conferring tolerance to various abiotic stresses (Ganesh et al., 2022; Vocciante et al., 2022). Unlike genetically modified organisms (GMOs), plant growth-promoting microorganism (PGPM)-based interventions offer a sustainable and environmentally friendly approach to improving crop resilience without genetic modifications or adverse environmental effects. This review highlights the significant contributions of microorganisms to sustainable crop production under challenging environmental conditions. By examining the mechanisms underlying PGPM-mediated salinity tolerance and their potential agricultural applications, we underscore the vital role of these microorganisms in addressing future agricultural challenges. We focus on PGPMs as a promising solution for overcoming the limitations of existing strategies in mitigating salinity stress. Harnessing the potential of PGPMs holds great promise for addressing the complex challenges posed by climate change and ensuring global food security amid increasing salinity stress. In this era of unprecedented threats to agriculture, it is imperative to develop innovative strategies to counteract these emerging challenges.

## 2 Salinity stress in plants and its impact on crop production and plant responses

Salinity, a major abiotic factor, severely affects the growth, development, and yield of various plants at different stages of their life (Khan et al., 2022). Soil salinization impacts agricultural productivity around the globe (Hu et al., 2022). Over 800 million hectares of irrigated land are impacted by soil salinity and are anticipated to be aggravated by both current irrigation practices and global climate change (Roy et al., 2014). The rising salinity in soils and water resources is contributed by natural incidents and/or human activities like irrigation water containing higher salts (Eswar et al., 2021). Saline soil with high Na^+^ negatively impacts soil–water and soil–air relationships, directly influencing plant growth and productivity (Rengasamy and Olsson, 1991; Dexter, 2004). Increasing salinity stress modifies soil texture, causing decreased porosity, which causes reduced water uptake by plants (Lu and Fricke, 2023). Salinity not only disrupts the soil’s physical structure but also significantly hampers the overall growth of plants, affecting shoots, roots, and reproductive organs. Salinity-induced modification of morphological, biochemical, and physiological processes in plants diminishes agricultural productivity. In addition, fluctuation in water dynamics, transpiration, nutritional equilibrium, stomatal conductance, and oxidative damage under salt stress collectively decrease crop yield. Moreover, salt stress hampers photosynthetic activity, impedes biomass accumulation, and disrupts source–sink dynamics, exerting a detrimental influence on yield-related variables and accelerating the senescence of essential organs (Khataar et al., 2018). Over time, the impact of salinity on plant productivity escalates, leading to economic losses and societal effects (Atta et al., 2023).

Plants can be categorized into two main groups based on their adaptive evolution: halophytes (salt-withstanding) and glycophytes (salt-sensitive) (Khan et al., 2022). The majority of crop plants belong to the glycophyte group and are adversely affected by elevated salt levels in the soil or irrigation water, impacting their growth, development, and yields (Shrivastava and Kumar, 2015). Salt-affected plants usually show dark green leaves, which are heavier and more succulent than typical plants of the same species (Amacher et al., 2000).

For instance, the impact of salinity on crop yields can be seen in specific examples. Beans experience no yield loss at an electrical conductivity of soil saturation extract (EC_e_) of 1.0 dS m^−1^, but show a 25% yield loss with EC_e_ = 2.3 dS m^−1^ and a 50% yield loss with EC_e_ = 3.6 dS m^−1^ (Amacher et al., 2000). Conversely, barley shows no yield loss with EC_e_ = 8 dS m^−1^, a 25% yield loss with EC_e_ = 13 dS m^−1^, and a 50% yield loss with EC_e_ = 17 dS m^−1^ (Amacher et al., 2000). It should also be noted that the salinity tolerance level of different cultivars of a specific species to salinity may show variation in responses and yields as observed in guar, alfalfa, and other crops (Sandhu et al., 2017; Kaundal et al., 2021; Sandhu et al., 2021, Sandhu et al, 2023).

Some stages of the plant growth are more susceptible to salinity stress than others (Sandhu and Kaundal, 2018). Several studies have illustrated that salinity stress leads to substantial yield losses in major crops during their reproductive stages. For example, salinity has been demonstrated to decrease plant height, the number of spikelets, spike length, grain weight, and overall yield (including both grain and straw) in wheat (Kalhoro et al., 2016). Additionally, the impact of salinity on grain yield depends on the stages of wheat development. For example, salinity diminishes grain yield by 39%, 24.3%, and 13.4% during anthesis, early booting, and mid-grain filling, respectively (Ashraf and Ashraf, 2016).

High soil salinity causes ionic toxicity and disrupts osmotic equilibrium in plants, causing plant nutrient imbalance and osmotic stress (Shrivastava and Kumar, 2015). Salt stress not only disrupts ionic homeostasis and enhances osmotic potential but also hinders several processes, including stomatal development, stomatal movement, and expansion of cells. In pea plants, it has been shown that the accumulation of ions in the apoplast contributes to cellular necrosis (Speer and Kaiser, 1991). Similarly, in rice, the salt accumulation in the apoplast disturbs cellular water relations, which leads to dehydration and subsequently causes wilting (Flowers et al., 1991). Yield losses in crops in response to salinity are primarily attributed to Na^+^ and Cl^−^. However, other ions also impact yield losses in crops. Toxicity impact varies among various ions and combinations of ions (Hawkins and Lewis, 1993; Sandhu et al., 2020). When the salinity level is low, it is easier for cellular machinery to transport salt ions into the vacuole to adjust to the flux of ions across the plasma membrane into the cell (Blumwald et al., 2000). In contrast, when the salinity level is high, the influx rates become elevated, disrupting the cellular ion homeostasis. It subsequently leads to the accumulation of cations like Na^+^, sometimes Mg^2+,^ and Ca^2+^, and anions like Cl^−^, PO_4_^3−^, and SO_4_^2−^ in the cytosol, stroma, and matrix rather than in the vacuole. Sodium ions impact plant development by not only delaying flowering but also hindering photosynthesis (Kim et al., 2007; Van Zelm et al., 2020). This impairment occurs through the inhibition of carbon-fixing enzymes and interference with proton-motive force. Furthermore, multiple studies have observed that the K^+^/Na^+^ ratio correlates with grain dry matter in wheat and other crops (Poustini and Siosemardeh, 2004; Kalhoro et al., 2016; El Sabagh et al., 2021).

Elevated levels of NaCl in the soil decrease water potential, consequently limiting the plant’s access to water from the soil; this, in turn, triggers osmotic stress in plants (Acosta-Motos et al., 2017). Ions like Na^+^ and Cl^−^ enter plants through the outer cells of the root (Van Zelm et al., 2020). Subsequently, these ions are transported from the xylem of roots to the shoots. The elevation of ions within plant cells initiates an ionic imbalance, leading to immediate osmotic stress, followed by ionic stress, subsequently ionic toxicity, and the generation of reactive oxygen species (ROS) (Munns and Tester, 2008). An increase in Na^+^ due to salinity inhibits biosynthesis and activity of diverse metabolic enzymes, prompts stomatal closure, and diminishes photosynthesis. In response to salinity-induced osmotic stress, plants synthesize various compatible osmoprotectants and solutes, including mannitol, inositol, trehalose, polyamines, glycine, betaine, and proline to mitigate the severity of the salinity stress (Munns and Tester, 2008; Park et al., 2016; Van Zelm et al., 2020).

**Figure 1:**
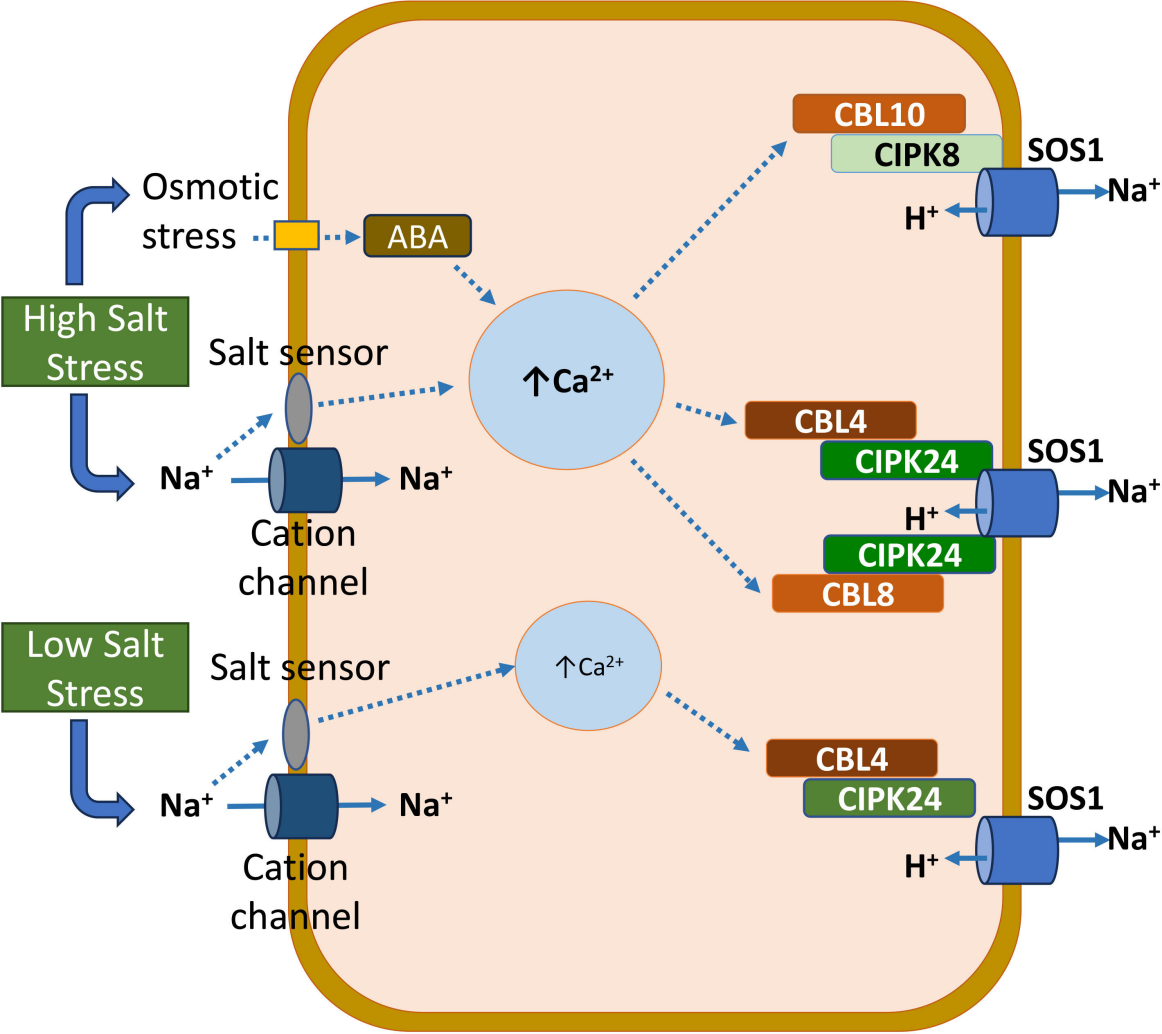
A model illustrating the role of the salt overly sensitive (SOS) pathway, including the SOS1, SOS2 (CIPK24), and SOS3 (CBL4) proteins, in maintaining Na^+^ homeostasis under low- and high-salinity stress in plants. In addition to SOS1, SOS2, and SOS3, CIPK8, CBL 8, and CBL10 contribute to Na^+^ homeostasis under high salt stress.

The intricate mechanisms through which plants perceive salts are not thoroughly comprehended. Salinity stress in plants triggers various signaling pathways, the combined effects of which confer salinity tolerance (Acharya et al., 2021). In response to salinity, MOCA1 (mono cation induced [Ca2^+^]_i_ increase 1), an extracellular salt sensor, detects Na^+^ and a few other monovalent cations (Jiang et al., 2019). MOCA1 synthesizes glycosyl inositol phosphorylceramide (GIPC) sphingolipids in the plasma membrane. GIPCs, with the ability to bind to monovalent cations like Na^+^, are implicated in the depolarization of cell-surface potential. It, in turn, triggers the opening of calcium-influx channels, leading to elevated intracellular Ca^2+^ levels. The activation of the salt overly sensitive (SOS) pathway follows the increase in intracellular Ca^2+^ (Zhu, 2002). Within this pathway, SOS3, upon binding with Ca^2+^, interacts with SOS2 and stimulates its kinase domain (Kaundal et al., 2022). Subsequently, SOS1 is phosphorylated by activated SOS2, facilitating the transport of Na^+^ from the interior to the exterior of the cell (**Figure 1**) (Quintero et al., 2011). The evidence described above indicates that both calcium and SOS signaling pathways are critical for plant’s salinity tolerance. In addition to SOS pathway components (SOS1, SOS2, and SOS3), CIPK8, CBL 8, and CBL10 contribute to Na homeostasis under high salt stress (**Figure 1**) (Acharya et al., 2024).

ROS are important secondary messengers in response to diverse stress signaling pathways, including salt stress (Ma et al., 2012). The excessive generation of ROS in response to salinity leads to oxidative stress, which, in turn, causes damage to proteins, membrane lipids, and nucleic acids (Ma et al., 2012). To protect cellular components and macromolecules from the detrimental effects of oxidative stress-mediated damage, plants engage in the synthesis of both non-enzymatic and enzymatic antioxidants. Plants synthesize various non-enzymatic antioxidants, including ascorbic acid (vitamin C), glutathione (GSH), and proline (Pro) (Gill and Tuteja, 2010). Enzymatic antioxidants are responsible for detoxifying ROS, encompassing superoxide dismutase (SOD), catalase (CAT), peroxidase (POX), and enzymes associated with the ascorbate (ASC)–glutathione cycle, such as monodehydroascorbate dehydrogenase (MDHAR), ASC peroxidase (APX), dehydroascorbate reductase (DHAR), and glutathione reductase (GR) (Gill and Tuteja, 2010; Foyer and Noctor, 2011).

Plant hormones are alternatively known as phytohormones, which play essential roles in plant growth and development and play critical roles in response to biotic and abiotic stress. In general, phytohormones are classified into two groups: auxin, brassinosteroids (BRs), cytokinins (CKs), gibberellins (GAs), and strigolactones (SLs) are known as plant growth hormones, and abscisic acid (ABA), ethylene (ET), jasmonic acid (JA), and salicylic acid (SA) are considered as plant stress hormones (Verma et al., 2016). The regulation of development, growth, and adaptation in plants under salinity stress is critically influenced by stress and growth hormones (Yu et al., 2020). A complex interplay occurs among plant stress hormones and plant growth hormones in response to salinity. These hormones play modulatory roles, engaging in complex crosstalk that significantly contributes to the plant growth adaptation during salinity stress (Yu et al., 2020). It should also be noted that the expression status of genes associated with phytohormone biosynthesis, transport, and signaling is an important determinant of salinity tolerance in plants (Acharya et al., 2022a, Acharya et al., 2022b).

## 3 Plant growth-promoting microbes to mitigate salinity stress

A diverse group of helpful microbes known as PGPMs inhabit the rhizoplane (root surface), rhizosphere (soil around roots), or endosphere (internal tissues). Generally, PGPMs include plant growth-promoting bacteria, rhizobia, and arbuscular mycorrhizal fungi. These microbes enhance plant growth in various ways, including producing indole-3-acetic acid (IAA), solubilizing phosphate for uptake, fixing nitrogen, producing beneficial enzymes like CAT (which helps to reduce oxidative stress), ACC deaminase (which reduces ET level that contributes to promote root growth), and producing siderophore (which chelates iron for plant use) (Mohanty et al., 2021). A detailed overview of the various ways PGPMs stimulate plant growth under non-stress conditions is thoroughly discussed in several recent reviews (Gahan and Schmalenberger, 2014; De Palma et al., 2022; Orozco-Mosqueda et al., 2023).

In this review, we focused on the role of PGPMs under salinity stress. Numerous research groups have discovered a wide array of PGPMs that alleviate salinity stress in plants. Various aspects of PGPM–plant interactions during salinity stress have been documented in previous research (Liu et al., 2022; Shrivastava and Kumar, 2015; Kaushal, 2020; Kumar et al., 2020; Mishra et al., 2021; Hoque et al., 2023; Mishra et al., 2023; Kumawat et al., 2024). In the following section, we categorize the various mechanisms through which PGPMs aid in mitigating salinity stress in plants.

### 3.1 Nutrient uptake and utilization

Essential nutrients are crucial for plant growth and yield, but their deficiency can negatively impact various aspects of plant development. During salinity conditions, elevated levels of sodium Na^+^ and Cl^−^ limit the uptake of macronutrients, including nitrogen (N), phosphorus (P), potassium (K), calcium (Ca), and magnesium (Mg) (Guo et al., 2020). It leads to a decreased availability of essential nutrients in plants, potentially triggering leaf senescence and inhibiting overall plant growth (Kumari et al., 2022).

Potassium-solubilizing bacteria (KSB) play a pivotal role in enhancing nutrient availability for plants, particularly in saline soils. Notable among these are bacterial species, such as *Pseudomonas* sp. and *Bacillus* sp., which can make K more accessible for plant uptake by solubilizing various silicate minerals (**Supplementary Table 1**) (Jaiswal et al., 2016; Vasanthi et al., 2018). Specifically, the PGPM strain *Burkholderia cepacia* SE4 has been shown to release K from soils, making it available to *Cucumis sativus* plants (Kang et al., 2014a). Moreover, the application of *Achromobacter piechaudii* to *Solanum lycopersicum* plants improved the uptake of K and P, while the application of salt-tolerant rhizobacteria, *Bacillus aquimaris*, in *Triticum aestivum* has been shown to enhance the uptake of K, P, and N in saline environments (Mayak et al., 2004; Upadhyay and Singh, 2015). Similarly, the application of *Azospirillum lipoferum* or *Azotobacter chroococcum* in *Zea mays* L under salinity enhanced K accumulation and provided salinity tolerance (Abdel Latef et al., 2020). In *Glycine max* seedlings subjected to salinity, inoculation with *B. firmus* (SW5) led to enhanced N and P accumulation and greater salinity tolerance, underscoring the significant role of *B. firmus* (SW5) in nutrient acquisition under stress conditions (El-Esawi et al., 2018). These findings suggest that salt-tolerant KSB can significantly enhance crop yields in saline soils.

Plant PGPMs also play a crucial role in increasing the accessibility of other essential minerals such as iron (Fe), zinc (Zn), and sulfur (S) to plants (**Supplementary Table 1**) (Gahan and Schmalenberger, 2014; Mishra et al., 2023). Iron is an essential micronutrient for plants as it is necessary for several metalloenzymes that are crucial in processes such as respiration and photosynthesis (Kobayashi et al., 2019). Salinity causes a deficiency of Fe that impacts plant growth, development, yield, and several other biological processes, including chlorophyll biosynthesis (Kobayashi et al., 2019). It has been documented that soil microbes play critical roles in accumulating Fe in roots and in transporting Fe in different plants (Masalha et al., 2000). Under conditions of low Fe availability, both microorganisms and plants produce siderophores—small organic molecules that selectively chelate ferric ions [Fe(III)], facilitating iron uptake (Ferreira et al., 2019; Timofeeva et al., 2022). The application of endophytic streptomyces has been demonstrated to significantly enhance the growth of mung bean and rice plants, leading to a notable increase in the biomass of both roots and shoots (Rungin et al., 2012). Furthermore, salt-tolerant siderophore-producing rhizobacteria (e.g., *Bacillus aryabhattai* MS3) have demonstrated the ability to promote plant growth in saline soils where Fe is limited (Sultana et al., 2020, Sultana et al., 2021). Therefore, siderophore-producing rhizobacteria are recognized as highly beneficial PGPMs, enabling plants to thrive in saline soils with limited iron availability (Ferreira et al., 2019).

Zinc (Zn) is a crucial plant micronutrient, essential for their development, growth, and yield (Saleem et al., 2022). Saline, sodic, and calcareous soil often cause Zn deficiency in plants (Tavallali et al., 2009; Daneshbakhsh et al., 2013). In the saline environment, the application of Zn is known to enhance sanity tolerance and stimulate proline metabolism (Mushtaq et al., 2023). Numerous studies have shown that PGPMs, including species such as *Trichoderma* sp., *Providencia* sp., *Anabaena* sp., and *Bacillus* sp., are capable of solubilizing Zn present in the soil (Upadhayay et al., 2022), which could be used for growth developments of plants including wheat (De Santiago et al., 2011).

Sulfur is a vital macronutrient crucial for the plant development and growth (Narayan et al., 2023). *P. putida* has been shown to play an important role in the S cycle in the conversion of organic S to an inorganic form that plants can uptake (Kertesz and Mirleau, 2004). Application of *P. putida*, *Pseudomonas fluorescens*, and *B. subtilis* provided salinity tolerance in soybean (Abulfaraj and Jalal, 2021). An *Enterobacter* sp., SA187, is known to promote alfalfa growth and yield in field conditions (De Zélicourt et al., 2018). Under salt stress, Arabidopsis plants showed symptoms resembling S starvation (De Zélicourt et al., 2018; Andrés-Barrao et al., 2021). However, when colonized with *Enterobacter* sp., SA187, these plants showed enhanced uptake of S and improved sulfur metabolism. This interaction also modulated the phytohormone signaling pathway and provided salinity tolerance (**Supplementary Table 1**) (De Zélicourt et al., 2018; Andrés-Barrao et al., 2021).

### 3.2 Synthesis of osmolytes and regulation

Salinity triggers osmotic stress in plants, leading them to produce various osmolytes that serve as osmoprotectants—like mannitol, inositol, trehalose, polyamines, glycine, proline, and betaine—to mitigate the severity of the salinity stress (Munns and Tester, 2008; Park et al., 2016; Van Zelm et al., 2020). In saline environments, PGPMs further support plants under osmotic stress by producing these osmoprotectants, thereby enhancing the plants’ tolerance to salinity (**Supplementary Table 1**). For example, inoculation of PGPMs, *P. fluorescens*, and *B. subtilis* enhanced proline accumulation in cucumber plants under salinity stress compared to non-inoculated plants (Saberi-Riseh et al., 2020). Similarly, the application of salt-tolerant *Stenotrophomonas maltophilia* BJ01 enhanced proline accumulation in peanut plants, providing salinity tolerance (Alexander et al., 2020). Additionally, *B. amyloliquefaciens* NBRI-SN13 enhanced proline and total sugar accumulation in rice seedlings, in contrast to non-inoculated seedlings (Tiwari et al., 2017).

In capsicum, the application of a salt-tolerant rhizobacteria, *B. fortis*, improved proline accumulation and conferred salinity tolerance (Yasin et al., 2018). Chickpea plants inoculated with *Azosprillum lipoferum* FK1 accumulated osmolytes like betaine, glycine, proline, and soluble sugars in response to salinity (El-Esawi et al., 2019). Further studies indicated that while salt treatment alone increased soluble sugars and proline content in maize plants, inoculation with *A. lipoferum* or *A. chroococcum* significantly boosted these levels, compared to plants treated with salt alone, enhancing maize growth under salinity (Abdel Latef et al., 2020). This suggests that the production of osmolytes and other components by these two PGPMs contributed to salinity tolerance and improved maize growth.

In soybeans, inoculation of *B. firmus* (SW5) not only increased the accumulation of osmoprotectants like glycine betaine and proline but also enhanced root architectural traits, including root length and volume, thereby improving salinity tolerance (El-Esawi et al., 2018). Moreover, an endophytic fungus, *Paecilomyces formosus*, known for producing Gas, provided salinity tolerance in cucumber by enhancing the accumulation of proline and other beneficial plant traits (Khan et al., 2012).

### 3.3 Enhancement of water transport

Aquaporins, integral membrane proteins from the major intrinsic protein (MIP) superfamily, form water-selective channels across membranes that play important roles in water transport and can also transport small neutral molecules (Kapilan et al., 2018). They play vital roles in cellular water transport as members of the plasma membrane intrinsic protein (PIP) and tonoplast intrinsic protein (TIP) families (Afzal et al., 2016). Aquaporins are key contributors to plant root hydraulic conductivity (Grondin et al., 2020). Expression of aquaporins is highly regulated by drought and salinity. An aquaporin gene *SpAQP1* of *Sesuvium portulacastrum*, a halophyte, was strongly induced in response to salt or drought treatment (Chang et al., 2016). Transgenic tobacco plants expressing *SpAQP1* demonstrated enhanced salt tolerance compared to wild-type and vector control plants, underscoring the role of aquaporin genes in salinity tolerance (Chang et al., 2016). In Arabidopsis, exposure to 100 mM NaCl leads to the downregulation of *PIP* and *TIP* aquaporin genes (Boursiac et al., 2005), a response also observed in other plants like cotton and tomato (Braz et al., 2019; Jia et al., 2020), significantly impacting root hydraulic conductivity (Siefritz et al., 2002). Conversely, the application of PGPMs has been shown to upregulate the expression of aquaporin genes, enhancing plant resilience to salinity. For instance, in maize, application of *Pantoea agglomerans* or *B. megaterium* upregulated aquaporin genes, improving root hydraulic conductivity and salinity tolerance (Marulanda et al., 2010; Gond et al., 2015). Similarly, barley seedlings treated with 200 mM NaCl exhibited reduced biomass and height alongside downregulated *HvPIP2;1* aquaporin gene expression (Zawoznik et al., 2011). However, inoculation with *Azospirillum brasilense* strain AZ39 induced *HvPIP2;1* expression, mitigating biomass and height reduction (Zawoznik et al., 2011). These observations suggest that *A. brasilense* strain AZ39 alleviates salinity stress possibly by upregulating the *HvPIP2;1* aquaporin gene, thereby enhancing root hair length, density, and improving water uptake. Furthermore, in response to salt stress, the mycorrhizae-mediated modulation of the expression of aquaporin genes has been reported in multiple plant species including *Phaseolus vulgaris*, *Lactuca sativa*, and *Robinia pseudoacacia* (Sharma et al., 2021). Mycorrhiza-mediated upregulation of aquaporin gene improved water status, K^+^/Na^+^ homeostasis, increased photosynthesis, enhanced expression of genes associated with ion homeostasis, including *SOS1* and *HKT1*, and alleviated salinity tolerance in black locust (*Robinia pseudoacacia*) (**Supplementary Table 1**) (Chen et al., 2017).

### 3.4 Regulation of ionic equilibrium

During salinity stress, toxic ions like Na^+^ and Cl^−^ increase in the cytosol, and excessive accumulation of these ions causes toxicity. Na^+^ not only imbalances the K^+^/Na^+^ ratio but also affects many physiological processes and functions of various proteins (Assaha et al., 2017). The SOS signaling pathway plays an important role to reduce Na^+^ inside of the cell, and that, in turn, helps maintain Na^+^ homeostasis (Park et al., 2016). Additionally, the high-affinity K^+^ transporter 1 (HKT1) contributes to Na^+^ homeostasis by removing Na^+^ from the xylem and sending Na^+^ back to the root (Kaundal et al., 2019). In rice, it has been shown that OsHKT1;4-mediated Na^+^ transport in stems plays a role in excluding Na^+^ from leaf blades during the reproductive growth stage in response to salt stress (Suzuki et al., 2016). Na^+^/H^+^ antiporters have been implicated in Na^+^ and K^+^ homeostasis and salt tolerance (Apse et al., 1999; Bassil et al., 2011). Moreover, the proton pump, AVP1, has been shown to play a role in salinity tolerance in different plants (Gaxiola et al., 2001; Lian et al., 2024).

Multiple PGPMs have been identified to induce expression of SOS pathway genes (**Supplementary Table 1**). Notably, volatile compounds produced by rhizobacteria *Alcaligenes faecalis* JBCS129 have been shown to upregulate expression of Arabidopsis *SOS1*, *HKT1*, *NHX1*, and *AVP1* under salt stress, assisting the plant in maintaining ion homeostasis during salinity stress (Bhattacharyya et al., 2015). The application of PGPM strain *Glutamicibacter* sp. YD01 in rice seedlings provided salinity tolerance by inducing expression of *OsHKT1* significantly and maintaining ion homeostasis (Ji et al., 2020). This strain also produces ACC (1-aminocyclopropane-1-carboxylate) deaminase and IAA, further supporting plant growth under stress.

In response to salinity, maize plants showed increased Na^+^, decreased K^+^, and reduced K^+^/Na^+^ ratio. However, salt-stressed maize plants inoculated with *A. lipoferum* or *A. chroococcum* showed reduced Na^+^, enhanced K^+^ accumulation, increased K^+^/Na^+^ ratio, and improved salinity tolerance, indicating that both *A. lipoferum* or *A. chroococcum* contribute to plant ion homeostasis in response to salinity (Abdel Latef et al., 2020). Similarly, inoculating white clover plant (*Trifolium repens*) with *A. brasilense* enhanced growth under salinity and reduced Na^+^ content, enhanced K^+^ content, and increased K^+^/Na^+^ ratio, suggesting that *A. brasilense* treatment contributes to ion homeostasis in plants under salinity (Khalid et al., 2017). Additionally, the application of *Variovorax paradoxus* 5C-2 to pea plants under salinity enhanced ion homeostasis by increasing K^+^ uptake, decreasing Na^+^ accumulation, and enhancing K^+^/Na^+^ ratio, leading to enhanced growth and salinity tolerance compared to uninoculated plants (Wang et al., 2016). Under salinity stress, rice inoculated with *C. albidum* strain SRV4 exhibited lower Na accumulation and higher K accumulation compared to non-inoculated plants (Vimal et al., 2019). This improved tolerance to salinity indicates the positive role of *C. albidum* in maintaining ion homeostasis during salt stress.

### 3.5 Production of antioxidants

Various plant enzymes, including APX, CAT, GR, POX, and SOD, exhibit antioxidant activity (Upadhyay et al., 2012). Many PGPMs have been shown to boost the activity of these antioxidant enzymes. For instance, *Piriformospora indica*, a root-colonizing basidiomycete fungus, promotes growth and provides resistance against mild salinity stress in barley by activating the antioxidative capacity through the glutathione–ascorbate cycle (Waller et al., 2005). Similarly, applying *A. lipoferum* or *A. chroococcum* to maize plants enhanced the activity of CAT and POX POD, showcasing their positive regulatory roles in salinity tolerance (Abdel Latef et al., 2020). In soybean seedlings, *B. firmus* (SW5) inoculation elevates the activity of APX, SOD, CAT, and POD, alongside reducing H_2_O_2_ levels, indicating its contribution to enhanced antioxidative capacity (El-Esawi et al., 2018). Inoculating okra seeds with ACC-producing *Enterobacter* sp. UPMR18 improved seed germination and seedling growth under salinity conditions (Habib et al., 2016). This was accompanied by heightened ROS-scavenging activity of enzymes like APX, CAT, and SOD, indicating that these ROS-scavenging enzymes play a beneficial role in enhancing salinity tolerance through the action of PGPMs (**Supplementary Table 1**). The application of *P. putida* H-2–3 showed a higher activity of SOD and improved soybean plant growth under salinity and drought (Kang et al., 2014b). Additionally, a GA-producing endophytic fungus, *P. formosus*, aids in salinity tolerance in cucumber by accumulating antioxidants, among other beneficial traits (Khan et al., 2012). Application of *Curtobacterium albidum* strain SRV4 in rice under salinity showed the higher activity of antioxidant enzymes, including CAT and SOD, and provided tolerance to salinity compared to non-inoculated plants (Vimal et al., 2019).

### 3.6 Phytohormone synthesis and regulation

Phytohormones play vital roles in regulating plant growth, development, and various physiological processes (Acharya and Assmann, 2009; Acharya et al., 2013; Miransari and Smith, 2014; Acharya et al., 2017). In response to salinity stress, various phytohormones, including auxins, CK, ET, and GAs, are critical in helping plant to adapt (Kaundal et al., 2021; Acharya et al., 2022a, Acharya et al., 2022b). Several PGPMs have been identified that produce and excrete hormones that plants can absorb through their roots, enhancing plant growth or regulating hormone balance to improve salinity responses (Backer et al., 2018). For instance, application of a halotolerant PGPM strain, *Glutamicibacter* sp. YD01, equipped with ACC deaminase, has been shown to provide salinity tolerance by reducing ET in rice seedlings (**Supplementary Table 1**) (Ji et al., 2020). Furthermore, PGPMs are known to synthesize some phytohormones like auxin, CK, ET, GA, and SA, modulating physiological activity through molecular responses (Orozco-Mosqueda et al., 2023). Additionally, many organic compounds produced by PGPMs are known to influence plant physiological activities, underlining the significant role these microorganisms play in enhancing plant health and stress resilience. The next section explores how PGPMs influence the regulation of various phytohormones.

#### 3.6.1 Auxin

Auxin, a crucial phytohormone, plays significant roles in plant growth and development, and particularly root development, including primary root elongation and lateral root initiation (Van Zelm et al., 2020). Additionally, auxin is vital in plant responses to salt stress; reduced auxin levels in roots under such conditions negatively impact root growth and architecture (Smolko et al., 2021). Specifically, salt stress, primarily through Na^+^, inhibits auxin-mediated primary root elongation and impedes auxin-mediated lateral root initiation, emergence, and elongation (Van Zelm et al., 2020). In response to salinity, the ABA concentration increases, which further inhibits lateral root emergence and elongation (Van Zelm et al., 2020). An increase in Na^+^ in roots reduces auxins and enhances ABA that, in turn, causes inhibition of lateral growth.

Many PGPMs are known to synthesize IAA, a physiologically active auxin, including species *Aeromonas veronii*, *A. brasilense*, *Enterobacter* sp., *Rhizobium leguminosarum, Actinobacteria*, *Frankia*, *Kitasatospora*, *Nocardia*, *Pseudomonas, Bacillus*, and *Streptomyces* (**Supplementary Table 1**) (Vessey, 2003; Kumar et al., 2020; Ganesh et al., 2022). The impact of the exogenous application of IAA is dependent on concentration; high concentrations accelerate the development of lateral roots and root hair formation while negatively affecting primary root growth (Vacheron et al., 2013). In contrast, a low dose of IAA may promote primary root growth (Vacheron et al., 2013). The application of PGPMs that produce auxin have been shown to induce plant growth by enhancing root growth and biomass (Backer et al., 2018). Additionally, many PGPMs indirectly influence auxin signaling pathways in plants. For example, some PGPMs with nitrite reductase activity, like *A. brasilense*, produce nitric oxide (NO), which is involved in lateral root development under stress conditions (**Supplementary Table 1**) (Wimalasekera and Scherer, 2022).

The uptake of IAA produced by PGPMs promotes primary and lateral root growth, as well as root hair proliferation, enabling plants to absorb more nutrients and minerals for improved growth and productivity. This indicates that the presence of PGPMs in soil, through the production of IAA, can significantly enhance plant growth compared to soils without PGPMs (Glick, 2014). Salt-tolerant rhizobacterial strains that produce auxin and proline mitigated salinity-induced growth inhibition of barley plants by regulating ion homeostasis and leaf water potential (Metoui Ben Mahmoud et al., 2020). A study showed that *Medicago truncatula* nodulated by an IAA-overproducing strain, *Sinorhizobium meliloti* RD64, showed improved tolerance to 300 mM NaCl (**Supplementary Table 1**) (Bianco and Defez, 2009). An IAA-producing PGPM strain, *C. albidum* SRV4, provided tolerance in rice by improving growth, improving K uptake, and boosting antioxidative enzymatic activities (Vimal et al., 2019). IAA-producing PGPM *Pseudomonas* sp. provides salinity tolerance in cotton (Egamberdieva et al., 2015). Three IAA-producing halotolerant PGPMs isolated from halophytes, *Micrococcus yunnanensis*, *Planococcus rifietoensis*, and *V. paradoxus*, have been shown to provide salinity tolerance in sugar beet (**Supplementary Table 1**) (Zhou et al., 2017).

#### 3.6.2 Gibberellins

GAs constitute a large group of phytohormones that positively regulate various aspects of plant growth, such as root and stem elongation, cell division, bolting, flowering, seed germination, and dormancy (Swain and Singh, 2005). DELLA (aspartic acid–glutamic acid–leucine–leucine–alanine) proteins, a sub-family of plant-specific GRAS (GIBBERILIC ACID INSENSITIVE, REPRESSOR OF *ga1–3*, and SCARECROW) transcriptional regulators, are critical components of the GA signaling pathway (Phokas and Coates, 2021). Abiotic stresses, such as salinity, are known to reduce GA levels, primarily by inhibiting the enzymes responsible for GA biosynthesis, highlighting the crucial role of GAs in plant stress resilience (Achard et al., 2006; Magome et al., 2008).

Many PGPMs are capable of producing GAs, which aid in plant growth enhancement (Backer et al., 2018). GA-producing bacteria, *Burkholdera cepacia* SE4, *Promicromonospora* sp. SE188, and *Acinetobacter calcoaceticus* SE370, provided salinity tolerance in cucumber plants (Kang et al., 2014a). Similarly, *Pseudomonas putida* H-2–3, another GA producer, improved soybean growth under salinity and drought (**Supplementary Table 1**) (Kang et al., 2014b). Further research has demonstrated that the application of the GA-producing endophytic fungus *Penicillium funiculosum* LHL06 can impart salt stress tolerance to soybean, by lowering plant levels of ABA and JA, and enhancing isoflavone biosynthesis (Khan et al., 2011). Additionally, the GA-producing endophytic fungus, *P. formosus*, provided salinity tolerance in cucumber by reducing stress hormone ABA and enhancing the accumulation of antioxidants and proline (Khan et al., 2012). A recent study shows that a GA-producing PGPM, *B. subtilis* ER-08 (isolated from a halotolerant plant), with multiple growth-promoting attributes enhanced the growth of fenugreek (*Trigonella foenum-graecum* L.) in response to salinity and drought stress (**Supplementary Table 1**) (Patel et al., 2023).

#### 3.6.3 Cytokinins

CKs have been identified as both positive and negative regulators in the context of salinity stress tolerance (Liu et al., 2020). For instance, increased CK levels during salinity stress have been observed in plants like Arabidopsis, rice, tomato, and apple. Notably, the *OsCKX2* knockout rice mutant, which has a higher level of CK content, shows higher salinity tolerance compared to wild type (Joshi et al., 2018). Additionally, the application of INCYDE, a CK degradation inhibitor, has been shown to increase salinity tolerance in tomatoes, underscoring CK’s beneficial role in salinity tolerance by suggesting that salt stress may reduce CK levels, thereby diminishing salinity tolerance (Aremu et al., 2014). Conversely, there are instances where increased CK levels have been associated with reduced salinity tolerance. Overproduction of CK in Arabidopsis showed reduced salinity tolerance (Wang et al., 2015). Furthermore, in Arabidopsis, CK negatively regulates the expression of *HKT1*, which is responsible for unloading Na^+^ from the root xylem, which, in turn, causes an increase of Na^+^ in the shoot (Mason et al., 2010). Additionally, reduced CK level due to increased degradation or reduced synthesis provided enhanced tolerance to salinity, including wheat and tomato (Avalbaev et al., 2016).

#### 3.6.4 Ethylene

It is well known that ET is one of the important phytohormones that play key roles in several plant physiological processes, including salinity stress (Riyazuddin et al., 2020). Salinity and other abiotic stresses increase ET content, causing the stunted growth of plants (Chen et al., 2021). ACC deaminase, an enzyme that hydrolyzes ACC, the immediate precursor of ET, plays a vital role in reducing ET levels, thereby aiding plant growth under stress conditions (Shahid et al., 2023). PGPMs utilize ACC deaminase to reduce ET levels, which, in turn, helps to reduce the stress level induced by salinity or other stresses (Glick et al., 2007; Orozco-Mosqueda et al., 2020). For instance, *P. fluorescens* strain TDK1, which produces ACC deaminase, has been shown to confer salinity tolerance and increase yield in peanuts (Saravanakumar and Samiyappan, 2007). Similarly, inoculation with *V. paradoxus* 5C-2, an ACC deaminase-producing PGPM, has provided salinity tolerance in *Pisum sativum* L. cv. Alderman, leading to increased biomass and enhanced photosynthetic activity (Wang et al., 2016). In addition, various studies have documented that rhizobacteria that have functional ACC deaminase provide tolerance in various crops, including *P. fluorescens* LSMR-29 and *E. hirae* LSMRS-7 in *Vigna radiata* (Kumawat et al., 2024), *Arthrobacter protophoramiae* in *P. sativum* (Barnawal et al., 2014), *P. fluorescens* NBRC 14160 and *B. megaterium* NBRC 15308 in wheat (Fathalla and Abd El-Mageed, 2020), *Glutamicibacter* sp. YD01 in rice seedlings (Ji et al., 2020), *Aneurinibacillus aneurinilyticus* and *Paenibacillus* sp. in French bean (Gupta and Pandey, 2019), *Bacillus* sp. PM31 in maize (Ali et al., 2023), and *Hartmannibacter diazotrophicus* E19^T^ in barley (**Supplementary Table 1**) (Suarez et al., 2015). Additionally, multiple species of ACC deaminase producing halotolerant PGPMs with additional growth-promoting properties isolated from halophytes, *P. rifietoensis*, *V. paradoxus*, and *M. yunnanensis*, have been shown to provide tolerance to salt stress in *Beta vulgaris* by reducing ET content (Zhou et al., 2017).

### 3.7 Biofilms

Biofilms are complex and structured communities of microorganisms, primarily bacteria that adhere to surfaces and are encased in a self-produced matrix of extracellular polymeric substances (EPS), comprising polysaccharides, proteins, nucleic acids, and other molecules (Di Martino, 2018). Biofilm-producing microorganisms gain a survival advantage in unfavorable conditions, including saline soils, where increased osmotic pressure could otherwise lead to cell death through cytoplasmic lysis. The ability to form biofilms equips these microorganisms with a protective mechanism against saline environments and other abiotic stresses, effectively serving as barriers that enable them to withstand and thrive under harsh conditions (Yin et al., 2019).

Halotolerant PGPMs thrive in saline environments, establishing themselves around the root zone, and promoting plant growth and development (Ahemad and Kibret, 2014). They produce various beneficial chemicals and growth regulators in the rhizosphere. Among these, certain halotolerant PGPM strains have been discovered to enhance salinity stress tolerance in plants. For example, two halotolerant biofilm-forming PGPM strains, AP6 and PB5, affiliated with *B. licheniformis* and *P. plecoglossicida*, respectively, were found to produce IAA and ACC deaminase (Yasmeen et al., 2020). These strains contributed to salinity tolerance and led to better growth and yield of sunflower plants than the non-inoculated plants. It demonstrates the multifaceted benefits of biofilm formation, including the production of IAA and ACC deaminase, which contribute to improved plant growth, productivity, and salinity tolerance (Yasmeen et al., 2020). Furthermore, wheat seedlings inoculated with exopolysaccharide-producing bacteria have been shown to stimulate growth and provide salinity tolerance by restricting Na^+^ influx (Ashraf et al., 2004). An exopolysaccharide-producing PGPM strain, *C. albidum* strain SRV4, provided tolerance to rice (Vimal et al., 2019).

## 4 Challenges in applying PGPMs in soil for improvement of crops

PGPMs have been extensively researched over the years, and many efforts have been made to leverage their potential for commercial use. Despite their significant promise for sustainable agriculture, their broad-scale implementation encounters various obstacles.

### 4.1 Inconsistent efficacy of PGPMs

PGPMs can be highly context-dependent, varying across different soil types and climates (Martínez-Viveros et al., 2010). One of the primary challenges in utilizing PGPMs is ensuring their survival and persistence in the soil environment. Soil conditions, such as temperature, pH, and the presence of competing microorganisms, can impact the viability of these microbes (Martínez-Viveros et al., 2010), making it difficult for farmers to predict and ensure positive outcomes from PGPM application, which, in turn, slows their widespread adoption in agriculture.

### 4.2 Specificity of action

While some PGPMs exhibit broad-spectrum benefits, many work optimally with specific plant species or crop cultivars (Dhawi and Hess, 2017; Pratush et al., 2018; Ma et al., 2020). Identifying the most effective strain for each crop–soil combination requires extensive testing, complicating large-scale implementation. Additionally, natural crop variability within a species further challenges finding a “one-size-fits-all” solution.

### 4.3 Issues in the development process

The development of PGPMs is based on screening assays in a laboratory setting, which measure specific PGPM activities such as IAA production, calcium phosphate solubilization, and siderophore production (Ganesh et al., 2022). However, the presence of these characteristics in microorganisms does not always guarantee effective PGPM function under field conditions. Conversely, microbes lacking these *in vitro* properties might possess alternative mechanisms for promoting plant growth, which are less well-understood. Because of this knowledge gap, such microbes risk being overlooked and discarded during the early stages of laboratory screening, potentially missing out on effective PGPM candidates (Cardinale et al., 2015).

### 4.4 Shelf life and viability

PGPMs are living entities with specific viability requirements, and owing to their structural and cellular composition, they have a relatively short shelf life (Arriel-Elias et al., 2018). Maintaining their effectiveness throughout production, storage, and application can be expensive, complex, and challenging, especially for small-scale farmers.

### 4.5 Regulatory hurdles and lack of standardization

Regulatory systems frequently lag scientific progress. Concerns about unintended environmental impacts and the complex nature of microbial communities can create regulatory hurdles, stalling commercialization efforts (Leggett et al., 2011). Additionally, the lack of consistent standardization in strain identification, characterization, and quality control for agricultural applications impedes broad adoption and undermines farmer confidence.

### 4.6 Economic benefit to farmers

A clear economic benefit demonstrated for farmers is crucial for widespread adoption. The microbes’ application method must align with the farmer’s equipment and agricultural practices. Factors like upfront costs, application complexity, and reliable performance data compared to conventional methods need careful consideration. Farmers commonly perceive PGPM formulations as costlier and less effective than chemical alternatives, which needs to be addressed.

### 4.7 Farmers’ risk-taking ability:

Crop producers usually depend solely on farming for their livelihood and sustenance, with little to no extra financial runway between two crops. With such financial constraints, farmers are unwilling to use nontraditional measures compared to tried-and-tested methods. This can pose a big hurdle in the widespread adoption of PGPMs.

More extensive work needs to be done by agricultural researchers and farmers to adopt PGPM formulations on a broader scale (Parnell et al., 2016). Educating farmers about the long-term benefits and building trust in PGPM technology is essential. Farmer education and awareness play a crucial role. Shifting from traditional practices to effectively utilizing PGPMs requires knowledge and training, which may be limited to certain regions. Addressing these challenges through continued research, improved formulations, streamlined regulations, and effective farmer education is crucial to unlocking the full potential of PGPMs and transforming agriculture toward a more sustainable future.

## 5 Conclusions

Salt stress disrupts various plant processes, including seed germination, seedling and root growth, development, early senescence, flowering, and yield, potentially leading to premature death. In saline environments, reduced water uptake causes osmotic stress due to changes in cell turgor. Plants synthesize osmoprotectants to cope, but these may be insufficient. Higher levels of Na^+^ and Cl^−^ lead to ionic stress and imbalance, specifically affecting the K^+^/Na^+^ ratio. Gene expression changes may enable some tolerance, depending on salinity levels and plant genetics. Salinity negatively impacts the acquisition of essential nutrients like N, P, and K and induces oxidative stress by increasing ROS accumulation, which can be harmful. Although plants produce antioxidants, these may not fully counteract oxidative stress. Stress hormones like ABA and ET increase under salinity, inhibiting growth, while growth hormones like auxins and GAs are inhibited, further negatively impacting plant growth.

**Figure 2:**
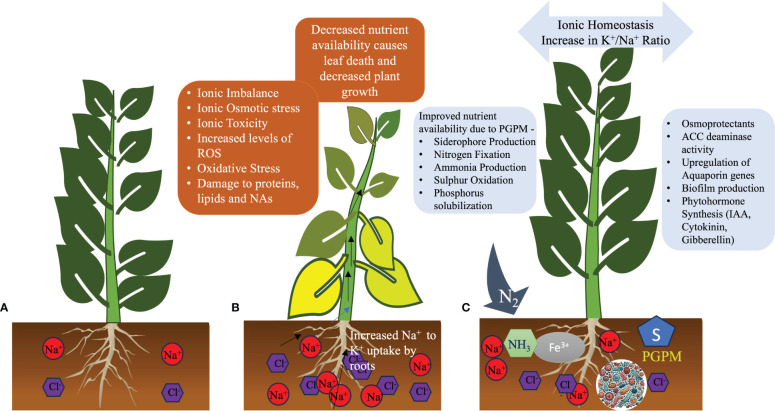
Roles of plant growth-promoting microorganisms (PGPMs) in enhancing salinity stress defense in plants. **(A)** A healthy plant in non-saline soil. **(B)** A plant facing saline conditions shows yellowing of leaves and stunted growth due to excessive ions in soil and tissues. **(C)** A plant in salt-affected soil treated with PGPMs regains health by mitigating the osmotic and ionic stresses induced by salinity.

In a saline environment, halotolerant PGPMs can play critical roles in improving plant growth (**Figure 2**). Their natural availability or supplementation of PGPMs alleviates the impact of salinity stress on plant development, growth, and yield by influencing various aspects of plant life. They modulate nutritional, physiological, biochemical, and molecular aspects of plant life. PGPMs enhance water uptake during salinity by upregulating the expression of aquaporin genes and additional mechanisms. Many PGPMs also enhance the accumulation of osmoprotectants in plants, thereby enhancing tolerance to salinity. They contribute to reducing the Na^+^ levels by upregulating genes that are involved in ion homeostasis, such as *SOS1* and *HKT1*, along with other genes playing roles in ion homeostasis. Halotolerant PGPMs also play critical roles in plant growth by enhancing the availability of essential nutrients, providing growth hormones like auxins and GAs, helping plants reduce stress hormones like ET through ACC deaminase, and enhancing the antioxidant capacity of plants. Employing PGPMs that produce ribosylated CK is beneficial, as this variant can move from the root to the shoot, promoting cell expansion and division without adversely impacting root growth, owing to its altered CK composition (Kudoyarova et al., 2019). Some PGPMs produce multiple hormones, enabling one to predict expected outcomes based on their respective functions. Specific combinations of PGPMs may be utilized according to the specific needs of a crop or plant to enhance salinity tolerance. Employing mathematical modeling, one can predict which combinations of PGPMs would be most effective in imparting salinity tolerance to a specific crop with known traits, including its tolerance level of salinity, as well as its physiological, biochemical, and molecular tolerance traits. Additionally, specific traits of PGPMs could be improved using gene editing technology tailored to the specific needs of a user. PGPMs present promising opportunities for sustainable agriculture by enhancing yields and resilience and decreasing dependence on harsh chemicals. Nevertheless, further efforts are needed to translate the potential observed in laboratory studies into broad-scale field applications.

## 6 Perspectives

Despite recent progress on PGPM-mediated salinity tolerance in plants, many questions remain unanswered. For instance, do PGPMs contribute to enhancing Na^+^ or salt-sensing mechanisms in plants? While literature suggests that many PGPMs provide general benefits like nutrient uptake, it is unclear if they are equally effective outside their natural range. Additionally, could some plant species be negatively impacted by specific strains of PGPMs?

Several reports indicate that PGPMs modulate gene expression in response to salinity. Do PGPMs induce epigenetic modifications in host plants under salt stress, affecting gene expression related to salinity tolerance? RNA-binding proteins (RBPs) are well-known regulators of gene expression at the post-transcriptional level. Given that PGPMs have been shown to provide salinity tolerance by regulating various genes, it would be highly interesting to investigate whether PGPMs specifically regulate gene expression through RBPs to promote salinity tolerance.

## Author contributions

BA: Conceptualization, Formal Analysis, Investigation, Validation, Visualization, Writing – original draft, Writing – review & editing. SG: Formal Analysis, Investigation, Validation, Writing – original draft, Writing – review & editing. AK: Conceptualization, Funding acquisition, Investigation, Resources, Supervision, Visualization, Writing – original draft, Writing – review & editing. DS: Conceptualization, Funding acquisition, Resources, Supervision, Visualization, Writing – original draft, Writing – review & editing, Investigation.
