# Supplementary material for: Strategies for combating plant salinity stress: the potential of plant growth-promoting microorganisms
Source: Front Plant Sci. 2024 Jul 15;15:1406913. doi: 10.3389/fpls.2024.1406913 (PMC11284086; doi:10.3389/fpls.2024.1406913)
Supplement: Supplementary file 1 [file Table_1.docx]

**Supplementary Table 1.** A summarized list of PGPMs that contribute to nutrient uptake; osmolyte synthesis and regulation; enhancement of water absorption; regulation of ionic equilibrium; production of antioxidants; and phytohormone synthesis and regulation; and may provide salt tolerance in plants. References are provided in the article.

| Trait | PGPR | Function/Contribution | Reference |
| --- | --- | --- | --- |
| Nutrient uptake and utilization | *Pseudomonas* sp. and *Bacillus* sp | Both PGPMs solubilize silicate. | Vasanthi et al., 2018 |
|  | *Burkholderia cepacia* SE4 | It solubilizes K in the soil, which helps *Cucumis sativus*. | Kang et al., 2014a |
|  | *Achromobacter piechaudii* | It contributes to the uptake of K and P in tomato plants. | Mayak et al., 2004 |
|  | *Bacillus aquimaris* | It contributes to the uptake of K and P in wheat. | Upadhyay and Singh, 2015 |
|  | *Azospirillum lipoferum* or *Azotobacter chroococcum* | Both PGPMs contributes to K accumulation in *Zea mays* L. | Abdel Latef et al., 2020 |
|  | *Bacillus firmus* (SW5) | It contributes to the enhanced accumulation of N and P in *Glycine max* seedlings. | El-Esawi et al., 2018 |
|  | Endophytic streptomyces, sp. GMKU 3100 | A siderophore-producing endophytic Streptomyces enhances the growth of rice and mung bean plants. | Rungin et al., 2012 |
|  | *Bacillus aryabhattai* MS3 | A salt-tolerant, siderophore-producing rhizobacterium promotes plant growth in saline soils where iron (Fe) is limited. | Sultana et al., 2020; Sultana et al., 2021 |
|  | *Trichoderma sp.*, *Providencia sp.*, *Anabaena sp.*, and *Bacillus* *sp.* | These PGPMs solubilize zinc and improve the growth of plants, including wheat. | Upadhayay et al., 2022 |
|  | *Pseudomonas putida* | It plays a role in the sulfur (S) cycle by converting organic sulfur (S) to an inorganic form that plants can uptake. | Kertesz and Mirleau, 2004 |
|  | *P. putida*, *Pseudomonas fluorescens*, and *Bacillus subtilis* | The application of these three strains provides salinity tolerance in soybean plants. | Abulfaraj and Jalal, 2021) |
|  | *Enterobacter* sp., SA187 | It provides salinity tolerance in alfalfa. | de Zélicourt et al., 2018; |
|  | *Enterobacter* sp., SA187 | It contributes to the uptake of sulfur (S) and plant sulfur metabolism. It modulates phytohormone signaling pathways and provides salinity tolerance. | de Zélicourt et al., 2018;  Andrés-Barrao et al., 2021 |
| Synthesis of osmolytes and regulation | *Pseudomonas fluorescens,* and *Bacillus subtilis* | Both PGPMs enhance proline accumulation in cucumber plants in response to salinity. | Saberi-Riseh et al., 2020 |
|  | Salt-tolerant *Stenotrophomonas maltophilia* BJ01 | It enhances the accumulation of proline in peanut plants and provides salinity tolerance. | Alexander et al., 2020 |
|  | *Bacillus amyloliquefaciens* NBRI-SN13 | It enhances the proline and total sugar accumulation in rice seedlings. | Tiwari et al., 2017 |
|  | Salt-tolerant rhizobacteria, *Bacillus fortis* | It provides salinity tolerance in Capsicum. | Yasin et al., 2018 |
|  | *Azosprillum lipoferum* FK1 | It enhances the accumulation of betaine, glycine, proline, and soluble sugars in chickpea plants. | El-Esawi et al., 2019 |
|  | *Azospirillum lipoferum* or *Azotobacter chroococcum* | Both PGPMs enhance the accumulation of soluble sugars and proline in maize plants compared to salt alone and enhance the growth of maize under salinity. | Abdel Latef et al., 2020 |
|  | *Bacillus firmus* (SW5) | It increases the accumulation of osmoprotectants, glycine betaine, and proline, enhances root architectural traits, and improves salinity tolerance in soybean. | El-Esawi et al., 2018 |
|  | Endophytic fungus, *Paecilomyces formosus* | Under salinity, it enhances proline accumulation and provides salinity tolerance in cucumber. | Khan et al., 2012 |
| Enhancement of water transport | *Pantoea agglomerans* or *Bacillus megaterium* | Either PGPM upregulates aquaporin genes, improves root hydraulic conductivity and enhances salinity tolerance in maize. | Marulanda et al., 2010;  Gond et al., 2015 |
|  | *Azospirillum brasilense* strain AZ39 | It enhances the expression of *HvPIP2;1* gene and mitigates biomass and height reduction in barley. | Zawoznik et al., 2011 |
|  | Mycorrhizae | It modulates the expression of aquaporin genes in *Phaseolus vulgaris*, *Lactuca sativa*, and *Robinia pseudoacacia.*  It upregulates aquaporin gene expression and improves water status and salinity tolerance. | Sharma et al., 2021  Chen et al., 2017 |
| Regulation of ionic equilibrium | *Alcaligenes faecalis* JBCS129 | It upregulates the expression of Arabidopsis *SOS1*, *HKT1*, *NHX1*, and *AVP1* under salt stress, and helps to maintain ion homeostasis. | Bhattacharyya et al., 2015 |
|  | *Glutamicibacter* *sp*. YD01 | It upregulates the expression of *HKT1,* maintains ion homeostasis, and provides salinity tolerance in rice seedlings. | Ji et al., 2020 |
|  | *Azospirillum lipoferum* or *Azotobacter chroococcum* | Both PGPMs reduce Na^+^, enhance K^+^ accumulation, increase K^+^/Na^+^ ratio, and improve salinity tolerance in maize. | Abdel Latef et al., 2020 |
|  | *Azospirillum brasilense* | It reduces Na^+^, enhances K^+^ accumulation, increases K^+^/Na^+^ ratio, and improves salinity tolerance in white clover (*Trifolium repens*). | Khalid et al., 2017 |
|  | *Variovorax paradoxus* 5C-2 | It enhances ion homeostasis by increasing K^+^ uptake, decreasing Na^+^ accumulation, and enhancing the K^+^/Na^+^ ratio. It also enhances the growth of pea plants and provides tolerance to salt. | Wang et al., 2016 |
|  | *C.* *albidum* strain SRV4 | Reduces the accumulation of Na, enhances the accumulation of K and provides tolerance salinity tolerance in rice. | Vimal et al., 2019 |
| Antioxidants | *Piriformospora indica,* a root-colonizing basidiomycete fungus | It promotes growth and provides resistance against mild salinity stress in barley by activating the antioxidative capacity through the glutathione–ascorbate cycle. | Waller et al., 2005 |
|  | *Azospirillum lipoferum* or *Azotobacter chroococcum* | Either PGPM enhances the activity of CAT and peroxidase POD, showcasing their positive regulatory roles in salinity tolerance in maize. | Abdel Latef et al., 2020 |
|  | *Bacillus firmus* (SW5) | It enhances the antioxidative capacity in soybean seedlings. | El-Esawi et al., 2018 |
|  | *Enterobacter sp.* UPMR18 | It enhances ROS-scavenging activity and provides salinity tolerance in okra. | Habib et al., 2016 |
|  | *Pseudomonas putida* H-2-3 | It enhances the activity of SOD and improves soybean plant growth under salinity and drought. | Kang et al., 2014b |
|  | GA-producing endophytic fungus *Paecilomyces formosus* | It aids in salinity tolerance in cucumber plants by accumulating antioxidants. | Khan et al., 2012 |
|  | *Curtobacterium albidum* strain SRV4 | It enhances the activity of antioxidant enzymes and provides salinity tolerance in rice. | Vimal et al., 2019 |
| Phytohormones | *Aeromonas veronii*, *Azospirillum brasilense*, *Enterobacter* sp., *Rhizobium leguminosarum, Actinobacteria*, *Frankia*, *Kitasatospora*, *Nocardia*, *Pseudomonas, Bacillus,* and *Streptomyces* | These PGPMs synthesize indole-3-acetic acid (IAA), a physiologically active auxin. | Vessey, 2003; Kumar et al., 2020;  Ganesh et al., 2022 |
|  | *Azospirillum brasilense* | It has nitrite reductase activity and produces NO that influences the auxin signaling pathway. | Wimalasekera and Scherer, 2022 |
|  | *Sinorhizobium meliloti* RD64 | It is an IAA (auxin) overproducing strain that provides salinity tolerance to *Medicago truncatula*. | Bianco and Defez, 2009 |
|  | *Curtobacterium albidum* SRV4 | It is an IAA-producing strain that provides tolerance in rice by improving growth, improving K uptake, and boosting antioxidative enzymatic activities. | Vimal et al., 2019 |
|  | *Pseudomonas* sp. | It is an IAA-producing PGPM that provides salinity tolerance in cotton. | Egamberdieva et al., 2015 |
|  | *Micrococcus yunnanensis*, *Planococcus rifietoensis*, and *Variovorax paradoxus* | These IAA-producing halotolerant PGPMs provide salinity tolerance in sugar beet. | Zhou et al., 2017 |
|  | Burkholdera cepacia SE4, Promicromonospora *sp.* SE188, and Acinetobacter calcoaceticus SE370 | These PGPMs produce GAs and provide salinity tolerance in cucumber plants. | Kang et al., 2014a |
|  | *Pseudomonas putida* H-2-3 | It is a GA producer that enhances soybean growth under salinity and drought. | Kang et al., 2014b |
|  | *Penicillium funiculosum* LHL06 | It is a GA-producing endophytic fungus that imparts salinity tolerance in soybean plants by lowering ABA and JA and enhancing isoflavone biosynthesis. | Khan et al., 2011 |
|  | *Paecilomyces formosus* | It is a GA-producing endophytic fungus that provides salinity tolerance in cucumber by reducing ABA and increasing the accumulation of antioxidants and proline. | Khan et al., 2012 |
|  | *Bacillus subtilis* ER-08 | It enhances the growth of fenugreek in response to salinity and drought stresses. | Patel et al., 2023 |
|  | *Glutamicibacter* sp. YD01 | It has ACC deaminase activity and provides salinity tolerance by reducing ethylene in rice seedlings. | Ji et al., 2020 |
|  | *Pseudomonas fluorescens* strain TDK1 | It has ACC deaminase activity, confers salinity tolerance, and increases yield in peanut plants. | Saravanakumar and Samiyappan, 2007 |
|  | *Variovorax paradoxus* 5C-2 | It is an ACC deaminase-producing PGPM that provides salinity tolerance in *Pisum sativum* L. cv. Alderman. | Wang et al., 2016 |
|  | *Pseudomonas fluorescens* LSMR-29 and *Enterococcus hirae* LSMRS-7 | Both are ACC deaminase-producing PGPMs that provide salinity tolerance in mung bean. | Kumawat et al., 2024 |
|  | *Arthrobacter protophoramiae* | It is an ACC deaminase-producing PGPM that provides salinity tolerance in *Pisum sativum*. | Barnawal et al., 2014 |
|  | *Pseudomonas fluorescens* NBRC 14160 and *Bacillus megaterium* NBRC 15308 | Both are ACC deaminase-producing PGPMs which provide salinity tolerance in wheat. | Fathalla and Abd El-Mageed, 2020 |
|  | *Glutamicibacter* sp. YD01 | It is an ACC deaminase-producing PGPM that provides salinity tolerance in rice. | Ji et al., 2020 |
|  | *Anurinibacillus Anurinilyticua* and *Paenibacillus* sp. | Both are ACC deaminase- producing PGPMs which provide salinity tolerance in French bean plants. | Gupta and Pandey, 2019 |
|  | *Bacillus* sp. PM31 | It is an ACC deaminase-producing PGPM that provides salinity tolerance in maize. | Ali et al., 2023 |
|  | Hartmannibacter diazotrophicus E19^T^ | It is an ACC deaminase-producing PGPM that provides salinity tolerance in barley. | Suarez et al., 2015 |
|  | *Planococcus rifietoensis*, *Variovorax paradoxus*, and *Micrococcus yunnanensis* | These ACC deaminase-producing PGPMs provide tolerance to salt stress in *Beta vulgaris* by reducing ethylene content. | Zhou et al., 2017 |
